# A New Year Message

**Published:** 1918-01-05

**Authors:** 


### The Hospital

The Workers' Newspaper of Administrative Medicine and Institutional Life, Administration, National Insurance and Health.

No. 1648, Vol. LXIII. SATURDAY, JANUARY 5, 1918 PRICE ONE PENNY

### A NEW YEAR MESSAGE.

The era of reconstruction which the opening of another year brings forcibly before the mind is one for which the hospitals are not unprepared, since the changes which the war itself has brought about came upon them with a rush, and their expansion has begun already. Their work must not so much grow as be fulfilled and maintained. The crisis of expansion is in large part behind them. With this reflection we can gauge the developments of the past year, for we have a test in the amount of consolidation that has been actually accomplished. New buildings, new institutions have sprung up in the hurried time behind us; how far have these begun to lay foundations which will make their continuance secure? On the one hand, we have observed the rapid increase in the Saturday Fund movement, and the enhanced sums which have been received from the new army of industrial recruits which these funds have embraced in diverse localities. On the other, in these centres the note of warning has been heard that, if this enhanced income is to be maintained in the prospective dislocation of industry, measures must be taken now, or the new funds may disappear as suddenly as they arose. If the problem ahead is the problem of maintenance, the difficult business of expansion has in part at least been mastered.

A fine thinker has remarked that "it is memory which makes us immortal," for it is memory which embalms the experience of the past. By it past and present are united, and from their union springs the child of memory, Hope. It will be noticed that Hope, like all the Christian virtues, is a gay one, for it can only arise in its buoyancy at the crisis of difficulty, and, like Faith and Courage, is never itself save in the presence of obstacles that would make the man without hope accept defeat. So that now when the strain of war makes victory depend upon endurance, we look to hope as the proof of our strength; for where hope is not endurance cannot be. A glance at the best administered hospitals shows that our leaders are full of it, for the men who are tireless are those whose exuberant activity can spring from hope and faith alone. The expansion and steady growth of the past year of hospital work at Cardiff are unparalleled instances of this. Colonel Bruce Vaughan has taken from Virgil the motto *potest quia posse videtur*, and has made the truth of it his own.

Another hopeful sign has been the new movement for the organisation of sectional subscriptions, whereby through a timely alliance employers' associations have undertaken the organisation of subscriptions at a settled rate among themselves, and have decided to replace a casual cheque by a proportional subscription. Just as the art of the association began with the workmen till it was adopted by the employers in their turn, so the organisation of employers' subscriptions has followed suit on the lines which the workers' funds have made familiar. By some such means as these the provinces are beginning to produce a system of finance suited to their requirements in the same way that from the peculiar circumstances of the capital was evolved King Edward's Hospital Fund. We mention the two together, because nothing better could express the difference between the needs and methods suited to London and those, on the other hand, which fit the provincial case. In both the question of maintenance has taken on a new significance, for the war has accustomed us to a new standard of accommodation, and laid upon the hospitals immensely larger duties than before. The consequence will be that institutional management will develop into a new and great profession, which will need a higher standard of *personnel* than has been requisite in the past. It was once true that anyone could be a hospital secretary. It is now the fact that only men of special endowment are fit for the higher posts. We foresee, therefore, that the choice between a medical superintendent and a trained lay administrator will cease to be an academic question. If the right recruits are not found in the ranks of the juniors the medical superintendent will be sought for much more generally than has been the case south of the Tweed.

We may notice, too, that every branch of institutional work is now developing its training-school, and that the domestic service in hospitals has begun to organise itself on school lines. It is, therefore, a question which will cease to raise a smile whether there should not also be a school for secretaries. We have only to observe the practice of America in this respect to see the example of what can be achieved on these lines. So far the work of administration has developed faster than the training of the men engaged upon it, and without the status that a fixed training alone can give it is difficult to attract the right class of candidates. In short, the voluntary hospitals have now begun to enter upon the full sphere of work which awaits them, and the future is as bright with promise as it is full of difficulty. In such a mood the year ahead can be awaited with the tense expectancy of starters for a race. The opportunities will be great for men who are capable of taking advantage of them. The prophet is he who can detect a tendency rather than one who guesses at future facts; the former is the quality of the statesman. On the statesman in ourselves we must rely if we are to face the future with the confidence that is born of strength; and the prospect is one which invites the best we have to give. The man of action requires no better call, for the hospitals which have triumphed over the difficulties of this war are capable of mastering all the tasks which the period of reconstruction, sooner or later, may lay upon them.

